# Identifying and enhancing risk thresholds in the detection of elder financial abuse: a signal detection analysis of professionals’ decision making

**DOI:** 10.1186/s12909-014-0268-z

**Published:** 2014-12-30

**Authors:** Priscilla Harries, Huiqin Yang, Miranda Davies, Mary Gilhooly, Kenneth Gilhooly, Carl Thompson

**Affiliations:** Department of Clinical Sciences, Brunel University, Mary Seacole Building, Uxbridge, Middlesex, England UB8 3PH UK; CRD, The University of York, Seebohm Rowntree Building, York, YO10 5DD UK; Brunel Institute for Ageing Studies, Department of Clinical Sciences, Brunel University, Mary Seacole Building, Uxbridge, Middlesex, England UB8 3PH UK; Department of Clinical Sciences, Brunel University, Mary Seacole Building, Uxbridge, Middlesex, England UB8 3PH UK; Health Services Research, Department of Health Sciences, The University of York, Seebohm Rowntree Building, Heslington, York, YO10 5DD UK

**Keywords:** Professional decision making, Elder financial abuse, Signal detection theory, ROC curve

## Abstract

**Background:**

Financial abuse of elders is an under acknowledged problem and professionals’ judgements contribute to both the prevalence of abuse and the ability to prevent and intervene. In the absence of a definitive “gold standard” for the judgement, it is desirable to try and bring novice professionals’ judgemental risk thresholds to the level of competent professionals as quickly and effectively as possible. This study aimed to test if a training intervention was able to bring novices’ risk thresholds for financial abuse in line with expert opinion.

**Methods:**

A signal detection analysis, within a randomised controlled trial of an educational intervention, was undertaken to examine the effect on the ability of novices to efficiently detect financial abuse. Novices (n = 154) and experts (n = 33) judged “certainty of risk” across 43 scenarios; whether a scenario constituted a case of financial abuse or not was a function of expert opinion.

Novices (n = 154) were randomised to receive either an on-line educational intervention to improve financial abuse detection (n = 78) or a control group (no on-line educational intervention, n = 76). Both groups examined 28 scenarios of abuse (11 “signal” scenarios of risk and 17 “noise” scenarios of no risk). After the intervention group had received the on-line training, both groups then examined 15 further scenarios (5 “signal” and 10 “noise” scenarios).

**Results:**

Experts were more certain than the novices, pre (Mean 70.61 vs. 58.04) and post intervention (Mean 70.84 vs. 63.04); and more consistent. The intervention group (mean 64.64) were more certain of abuse post-intervention than the control group (mean 61.41, p = 0.02). Signal detection analysis of sensitivity (A´) and bias (C) revealed that this was due to the intervention shifting the novices’ tendency towards saying “at risk” (C post intervention -.34) and away from their pre intervention levels of bias (C-.12). Receiver operating curves revealed more efficient judgments in the intervention group.

**Conclusion:**

An educational intervention can improve judgements of financial abuse amongst novice professionals.

## Background

Financial abuse is defined as ‘theft, fraud, exploitation, pressure in connection with wills, property or inheritance or financial transactions, or the misuse or misappropriation of property, possessions or benefits’ [1]. It is one of the most prevalent forms of abuse of older people [2,3]. It is also one of the most challenging areas of professional judgement and decision making in social care [4].

Because financial abuse is hard to detect, current prevalence rates of between 0.7% and 14.4% probably represent an underestimate [5]. It has been shown that victims of mass fraudulent “scams” alone cost victims £3.5 billion a year in the UK [6]. However, not all scams are reported. In Queensland, Australia, $14 million was exploited from elders in 2007/8; however as only 14% of reported cases included the financial amount that the older person had lost, $97 million represents a more realistic figure for Queensland, Australia [7].

Older people tend not to report financial abuse; in some instances they may not be aware that they are being abused. Even when aware, they may choose not to report the abuse, especially if they have been abused by a family member or have been duped in a scam [8]. In addition, older people with cognitive deficits, although often targeted by abusers [9], tend to be excluded from prevalence surveys due to the complexity of organising consent to participate [2].

Protecting the financial assets of the victim, reducing the risks of subsequent physical harm and associated loss of independence [10], requires early intervention. Mandatory reporting has been introduced in some parts of the United States to try to ensure suspected abuse is investigated [11]. Raising suspicions of abuse is not without risk however. A professional may not wish to report suspected abuse and risk the chance that they may be mistaken. A mistaken claim can lead to reprimands from employers and rejection by clients. Indeed nurses have reported reluctance to report abuse, for fear of recrimination [12]. In situations where the suspected abuser is also the carer, professionals may feel cautious about reporting their suspicions: instigating safeguarding procedures may jeopardise the support systems surrounding the person being abused and possibly cause more harm than good, especially if suspicions turn out to be unfounded. Professional judgement in this area involves balancing the need to risk false alarms with the risk of allowing abuse to continue unchecked. It is complex and an exemplar of the kind of “irreducible uncertainty” in social policy identified by Hammond [13].

As well as these “internal” qualities in decision makers, other contextual factors also impact on the effectiveness of strategies for reducing abuse. Although a multi-agency approach is advocated [1] professionals are often concerned about sharing information across and within due to data protection issues and lack of familiarity with communications systems in other sectors. In one study of adult safeguarding co-ordinators’ experiences [14], financial professionals were unwilling to become involved in addressing suspected financial abuse, or to share information with safeguarding teams - presenting a barrier to effective safeguarding.

Underestimated prevalence means that professionals tend not to consider financial abuse routinely [15]. They have difficulty recognising abuse and even when it is identified they do not always report it [16]. They are not fully aware of the risk factors or warning signs of abuse and without training it has been shown that they struggle to identify this underappreciated but prevalent problem [17]. Very thorough and useful research has been undertaken to examine the factors that might influence professional recognition and reporting of elder abuse as a whole [18], but more specific research is needed to examine those factors linked to the most prevalent form of elder abuse: elder financial abuse. Educational programmes can be an effective means of increasing awareness, collaboration and improved detection making capacity in this domain [19].

Professionals working in more senior posts have been shown to be more able to recognise signs of elder abuse than junior colleagues. For example, Kitchen et al. [12] demonstrated that senior nurses were able to take more appropriate action in cases of elder physical abuse than junior nurses. Scores were measured by comparing nurses’ self-reported actions in response to case vignettes against a model answer reflecting the “correct” policy. Senior nurses had a more appropriate threshold for triggering interventions, whilst junior nurses’ threshold for taking action was too low. However, these results only held where the vignette reported definite abuse. Where cases were less clear-cut (i.e. more uncertain), no differences were found in action capacity between junior and senior nurses. The authors suggested that this may be because nurses are ill equipped to deal with such sensitive and complex decisions and do not know the appropriate procedures for reporting. They highlighted the need for comprehensive training to be developed to assist junior clinicians to develop more appropriate thresholds for abuse detection and prevention.

At the heart of decisions to prevent and counter elder abuse are the judgements of health and social care professionals. There is evidence that various types of information impact on the judgements made by these professional groups. For example, Davies et al. [20] used social judgement analysis to reveal that the nature of the financial problem and the clients’ cognitive capacity both significantly influenced professionals’ certainty of abuse and the likelihood of taking action. Professionals were found to be most likely to act in response to particular kinds of financial problems, such as family members misusing Power of Attorney, or where older people were thought to be the target of rogue traders. Significant risk was also identified in cases where older people had limited mental capacity; the more limited their capacity the higher the perceived risk of abuse. The findings were used to develop an on-line educational intervention to improve novices’ ability to identify elder financial abuse. A trial was undertaken to test the effectiveness of the intervention with the mean expert risk score of each case being used as a reference standard against which novices’ performance was measured. The intervention was found to be effective in enhancing the novices’ capacity to detect and take action in cases of elder financial abuse [21].

Davis et al. [20] did not look at the thresholds or “tipping points” above which novices’ classified evidence as indicative of abuse. These relationships matter, as professionals lacking experience are not usually well “calibrated”; they need to be more sensitive to recognising abuse [22]. Recognising the uncertainty in a judgement or decision is a key driver for information acquisition [23] and empirically the evidence surrounding professionals’ confidence and their related judgement performance [24-27] suggests room for improvement. In Davies’ study, certainty of abuse was shown to be strongly correlated with the decision to take action to prevent abuse [20]. Interventions that can heighten sensitivity to recognition of abuse, through a lowering of risk threshold could be expected to lead to a more appropriate, heightened action response.

Research has yet to examine whether those less experienced have a different threshold level from more experienced professionals and if so whether they can be trained to alter their threshold to obtain a closer match to those with more experience. If they can shift their threshold to identify more cases, ideally while not increasing the number of false positives cases, their ability to recognise elder financial abuse will have become more effective.

Successfully intervening in cases of elder financial abuse then requires the ability to, appropriately, manage the identification of risk in uncertain situations and to separate out and pay attention to the truly relevant in a situation whilst discounting the irrelevant. At the same time the professional needs to deploy these skills in ways that minimise incorrect allegations and maximise the probability of alleging abuse when it is – in truth – present.

In this paper we address the following research questions:

1. Do novice and expert thresholds for detection differ from each other when examining case scenarios of elder financial abuse?
2. Does training bring novice detection of financial abuse risk in line with expert opinion?
3. Does training improve novices’ ability to identify more cases of abuse (hits) without increasing the number of false positives?

## Methods

The RCT (“Educating novice practitioners to detect financial abuse”; ESRC grant number RES-189-25-0334), from which the present analysis is drawn, aimed to test the effectiveness of a decision-training intervention on the capacity to detect and prevent elder financial abuse. A parallel-group, randomised controlled trial was conducted using a judgement analysis approach [21]. The intervention group was provided with training after baseline testing, whereas the control group were purely given instructions to continue with the task. The intervention comprised of written and graphical descriptions of an expert consensus standard explaining how case information should be used to detect and prevent elder financial abuse. Participants’ ratings of certainty of abuse (detection) and selected actions (prevention) were correlated with the experts’ ratings of the same cases at each stage of testing, as well as examining effect on mean group scores and cue use. The results of this study indicated that the decision-training aid has a positive effect on decision capacity. Further details of the RCT are given in the following sections as needed.

### Signal Detection – A theoretical framework for exploring judgements of elder financial abuse

Signal detection theory (SDT) approaches offers a theoretical framework for exploring decision thresholds and the ability to separate risk (signals) from the noise in situations of possible abuse. SDT has been applied to many areas of judgement and decision making [28] since its initial application to communication and psychology [29]. Examples include distinguishing between truth and lies and medical diagnosis and radiological interpretation [30] and decision making in cases of child abuse [31].The methodology has also been applied to examine the assessment of risk (of patients’ critical events) by nurses [32] and to explore the impact of enhanced “realism” in clinical simulation on the ability to detect risk signals [33].

In this paper we expand on an earlier social judgement analysis [21]. Combining SDT and social judgement analysis has proved useful previously: Cheyne et al. [34] compared the decision to transfer of women from rural led maternity care to acute services by midwifes and obstetricians. Social judgement analysis illustrated variables influencing the decision to transfer and the importance attached to them. Signal detection analysis explored the performance of judgement polices for identifying high and low risks cases, and compared the thresholds for action between professionals. Whilst professionals used similar variables, their personal thresholds for deciding to transfer a woman varied significantly.

To the best of our knowledge, SDT approaches have not been used before to explore suspected elder financial abuse. We are interested in examining how thresholds compare between novices and experienced professionals; as elder financial abuse is generally under reported it is proposed that experienced professionals would have a lower threshold for detection of abuse than novices; experts would therefore identify more cases of abuse from the case set presented than novices. Crucially – because they have had the opportunity to “learn” from experience – it is reasonable to assume that this ability to identify more cases of abuse would not be at the expense of incorrectly alleging abuse. Because it is often presumed that training can be used to bring novices’ performance closer that that of experienced practitioners, we are also interested in whether novices can be “trained” to bring their threshold down to a level that allows them to identify cases of abuse whilst not increasing the number of incorrect allegations. This effect would need to tested against a control; the control group would also be asked to judge the same scenarios but without the opportunity for training. If novices can be helped to identify more cases of actual abuse (actual cases being those which experienced professionals identify), then their skills will have been enhanced. We will focus on clinicians’ decision making (health and social care professionals) because they are well placed to detect elder financial abuse [17].

To illustrate the role of SDT for unpacking judgements of financial abuse of elders, consider a health or social care professional judging the likelihood of financial abuse of an older adult with whom they are working (“yes” they are at risk or “no” they are not at risk). Each older adult has a true, but unknown and uncertain, risk of abuse. Thus there are four possible outcomes associated with the judgement (Table 1):

**Table 1:** **The four possible outcomes from a “yes/no” signal detection task**

Judged level of financial abuse		True level of financial abuse risk	
		At Risk	Not At Risk
	At Risk	True positive/HIT	False positive/FALSE ALARM
		Correct outcome	Incorrect outcome
	Not At Risk	False negative/MISS	True negative/CORRECT REJECTION Correct outcome
		Incorrect outcome	

**Figure 1:**
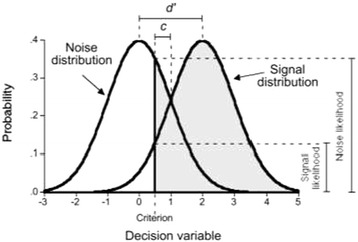
**Distribution of the decision variable across noise and signal scenarios, showing d´, c, and the likelihoods on which β is based.**

As Table 1 illustrates, judgments in which a professional correctly indicates that an elderly person is at risk (when in truth they are indeed at risk) can be termed ‘hits’. Incorrectly indicating a patient is at risk (when in truth they are not) represents a ‘false alarm’. Thus, for this yes/no (risk/not at risk) judgement the hit rate (probability of correctly classifying an elder as at risk) and the false alarm rate (the probability of incorrectly classifying an elder as at risk) describe judgement performance [28]. Where a professional makes judgements on elders who in truth (a state which is unknown to the professional) are “at risk” these are termed “signal” cases. Conversely, judgements on elderly people who are not “at risk” (again, a state that is unknown to the professional) represent “noise” or “no signal” scenarios. When making judgements, individuals draw on internalised rules [28]. These rules are unique to each person but common examples include the intuitive feeling that, “something is not right” [35], or perhaps a systematic unwillingness to make a judgement call of heightened risk of financial abuse for fear of, “getting it wrong” or setting off punitive investigative process. Often, these applied rules can efficiently separate signal scenarios from noise scenarios [36,37]. Over many judgments, the information present in signal and noise scenarios will differ and professionals should amend their judgement rules accordingly. Thus, for multiple signal cases there will be a distribution of hit and false alarm rates, with an equivalent distribution for noise cases. Reducing the overlap between the signal and the noise distribution would increase the hit rate whilst decreasing the false-alarm rate. Figure 1 illustrates the idea of signal and noise distributions arising from multiple cases of the same judgment.

Figure 1 illustrates two important concepts that have been operationalised from the hit and false alarm rates (see data analysis section for means of classification of hits and false alarms). Firstly, each professional will have a tendency towards classifying people as “at risk” or “not at risk”; this is referred to as beta, or β. When professionals favour neither the ‘yes’ response nor the “no” response, beta equals one. A value of β less than one indicates a bias toward responding ‘yes’, whilst a value of β greater than one indicates a bias toward responding “no”. Furthermore, each professional’s signal and noise distributions will overlap; the greater the overlap (or the smaller the distance between the peaks of their distributions) the lower the ability of the professional to separate signals (risk) from noise (no risk); a distance between the peaks of signal and noise distributions referred to as *d´* prime or *d*´. This *d*´evaluates the distance between the means of signal and noise scenarios in standard deviation unit. A value of zero for *d*´indicates an inability of the professional to distinguish signals from noise, whilst a larger value signifies his/her greater ability to distinguish signals from noise [28].

For some judgements the distributions of *d*´ and β are not normally distributed. Accordingly, two non-parametric measures of *d*´ and β exist: A´ (A prime) and C [28]. A´ ranges from .5, representing a person who cannot distinguish signals (“risk of abuse”) from noise (“no abuse”), to 1, representing “perfect” performance. C is the distance between the decision criterion and the (neutral) point at which neither a signal or noise response is favoured (i.e. where β = 1) for C this is equal to 0 [28]. The degree of deviation from this neutral point is measured in standard deviation units and so positive units indicate a tendency to say no (not at risk) and negative values indicate a tendency to say yes (at risk).

### “Signal” scenarios

In order to identify cases representative of financial abuse, we used experienced health and social care professionals’ decisions (n = 33) on a set of 43 scenarios used in a previous study [38]. The professionals (n = 33) ranged from 23 to 61 years old, with a mean age of 46 years. The sample included 26 females (79%), and 7 males (21%). Twenty-six participants (79%) recorded their ethnicity as ‘White – Welsh/English/Scottish/Northern Irish/British’. They had been in their current job for between 1 year and 20 years, with a mean of 7 years. They had been in the profession for between 2 and 35 years, with a mean of 16 years. The expert status of the professionals group could be questioned as some had limited experience, short time current experience in post, etc. It is also quite possible that some of the more experienced professionals had not become experts; experience is no guarantee of expertise [38]. The means by which expert status can be assured is difficult where no gold standard criterion exists; length of experience, level of education and work position held are no guarantee of expert ability [39]. However these 33 “experts” had been selected from a larger sample of 152 social care and health professionals who took part in a piece of research conducted by Davies et al. [38] to determine judgement policies used by professionals in cases of suspected of elder financial abuse. The means by which the 33 experts had been selected was based on an empirical measurement of their expertise ability. This was a bootstrap approach in which the individual’s own decisions were used to validate or invalidate their status as experts. This approach, known as the CWS approach, uses inconsistency and discrimination to determine expertise capacity and has been shown as a valid expert index across a range of domains [39]. The 33 expert professionals were therefore selected on the basis of being those with the highest CWS indices, i.e. they had a low level of judgement inconsistency and a high ability to modify judgements in line with changing information; essential characteristics of expert decision makers [39] (see Davies, 2011 for further details). The expert status is therefore assured as far as is feasible for a domain where no external criterion exists, against which to measure expert ability.

Each expert recorded their “certainty of abuse” for each scenario a visual analogue scale ranging from ‘Certain abuse is not occurring’, to ‘Certain abuse is occurring’ (0–100). These anchors were developed with experts in the field from health, social care and charitable sectors; groups who were dealing with these issues on a regular basis. The labels could have anchored in other ways for example the concepts of “very low risk” and “very high risk” could have been used; this would present the judgement range as being a spectrum between the two extremes; future researchers may like to consider this. In our study, a certainty of abuse score for each trial was recorded for each of the 33 experts for each of the scenarios. The mean certainty of abuse and 95% confidence intervals for the expert sample were calculated (see Table 2). Those scenarios where the experts’ average certainty was greater than the 95% upper confidence limit (73.4) were deemed “signal” trials: i.e. if we undertook the study repeatedly, with different experts, 97.5% of the time they would assign an overall mean level of certainty lower than 73.4.

**Table 2:** **Experts’ mean certainty of abuse, standard deviation and 95% confidence intervals**

Mean*	**N (scenarios)**	**SD**	**95% confidence interval**
70.61	28	8.63	67.97 – **73.4**

*range 0–100, higher score indicates more certainty of risk of abuse.

The number of scenarios to be used with the novices as the ‘before’ and ‘after’ training scenarios were calculated using the ratio calculations detailed in the methododological literature on judgement analysis [40]. Once these two sets of scenarios had been identified, the signal/no signal scenarios were calculated. Those scenarios in which the mean was less than or equal to the 95% upper confidence interval bound constituted “no signal” scenarios. Thus, a signal trial was one in which the experts collectively viewed the risk of abuse as significantly higher than average.

Applying this rule yielded 11 signal and 17 no signal scenarios in the cases the novices would judge as pre intervention scenarios and 5 signal and 10 no signal scenarios in the cases which the novices would judge as post intervention scenarios (see below for trial design).

### Capturing novice signal detection

An a priori sample size calculation was undertaken: in order to identify a medium effect (*r* = .3) it was calculated that 48 participants would be needed for both the intervention and control group when using an α – level of .05 and .8 power. Novice professionals (students) from 11 pre-registration clinical programmes across eight universities in the south of England were non-randomly selected to take part in the trial [20,21]. A randomisation table was created by MD in Excel. The Excel RANDBETWEEN (0,1) function was used to allocate each of the participants to one of two groups - either intervention or control - and participating students were then given an individual password. The intervention group judged 28 scenarios prior to receiving an educational intervention that was aimed at improving recognition of financial abuse in elders and 15 scenarios after receiving the intervention. The control group did not receive any training at any stage , however they did judge the same scenarios as the intervention group.

### Training intervention

The intervention outlined to participants the way in which experts chose to weight the relative importance of the cues and the content of the cues included in the case scenarios, thus highlighting the pertinent factors to consider in instances of suspected financial abuse. Within the scenarios, there were seven types of cues. These comprised the identifier of the abuse (e.g. professional, family, friend, older person); the financial problem (e.g. stealing, anomalies in finances, unknown befrienders, misuses of power of attorney and rogue traders); and the mental capacity and physical capacity of the individual. Additionally, age, gender and living circumstances of the individual were included so as to contextualise each of the scenarios.

The information that was provided in the training educated participants on how to utilise the cue information when making decisions and how to interpret the content of these cues. Particular emphasis was placed upon identifying the nature of the financial problem and the mental capacity of the individual, which were regarded as the two most significant cues in aiding detection of financial abuse. Graphical and descriptive information was developed and tested to establish ease of understanding and to ensure comprehensiveness of optimal knowledge transfer. The test site for the decision training aid was hosted online. It was not possible to determine to what extent the intervention group fully engaged in the use of online training resource as each participant did this remotely at a time and place of their choosing. A few mechanisms were included in the design to reduce the chance of disengagement for example they did have to scroll down and press on the continue button at the end of each page of the training information before proceeding to the next page but this was no guarantee that they had thoroughly read and understood the training information. The only way by which engagement could be assumed would be if there was a positive impact of the exposure on the novice decision makers’ judgements.

As the novices made judgements of risk of abuse (“certainty of abuse”) on the same set of 43 scenarios that the experienced clinicians had judged, mean level of certainty and a 95% upper confidence interval limit could be computed for the control and intervention groups for both the pre and post intervention sets of scenarios. The evaluations of the second set of scenarios were undertaken immediately after the novices had finished viewing the intervention information. Pilot testing identified that full task could be completed by novices and experts in under an hour which was deemed manageable by the pilot participants.

As with the experienced clinicians, where a novice judged a trial as more certain (of abuse) than the upper 95% confidence limit, this indicated that, in their judgement, the elderly person was significantly more at risk of financial abuse. Thus, the decision variable was the sense of certainty that the expert/novice felt when confronted with information that may indicate financial abuse and the criterion (the value that defines sufficiently high certainty to warrant a judgement call that this might be abuse) was point beyond which action is warranted.

### Ethical approval

Brunel University London’s School of Health Sciences and Social Care Research Ethics committee granted ethical approval for the research.

### Data analysis

Data were analysed as a yes/no signal detection task [28]. Hits and false alarms were calculated for each novice for each trial in the pre and post intervention trial sets. For the signal scenarios a novice level of certainty significantly higher than average constituted a *hit*. On the noise scenarios a novice level of certainty significantly higher than average constituted a *false alarm*.

Hit *(H)* and false alarm *(F)* rates were calculated for each novice. *H* was found by dividing the hits for each novice by the number of signal scenarios. *F* was calculated by dividing the number of false alarms by the total number of noise scenarios. Z scores for *H* and *F* and signal detection measures were computed using the formulae and SPSS syntax outlined by Stanislaw and Todorov [28].

After calculating mean scores for certainty, C and A´ for both intervention and control groups at baseline and immediately after the training intervention, assumptions of normality were tested (using the Shapiro-Wilk test in SPSS ver 21.0). Distributions were non-normal in one or other of the groups for certainty, H, F, *d*´, β. Thus A´and C and the non-parametric test (Mann–Whitney U test) were used to test the significance level of the difference between groups. All analysis was undertaken in SPSS version 21.

ROC curves for the intervention and control groups were calculated using the procedures outlined in Zhang and Mueller [40], these procedures thereby correcting for differences in distributions, false alarm and hit rates. Zhang and Mueller’s [41] procedures use two slightly different (non parametric) measures of sensitivity and bias to the more commonly reported measures of A´and C [28]. Their measure of sensitivity is “A” and bias (“b”); interpretation is identical to A´and C and readers are directed to Zhang and Mueller [41] for the necessary computational procedures.

## Results

**Figure 2:**
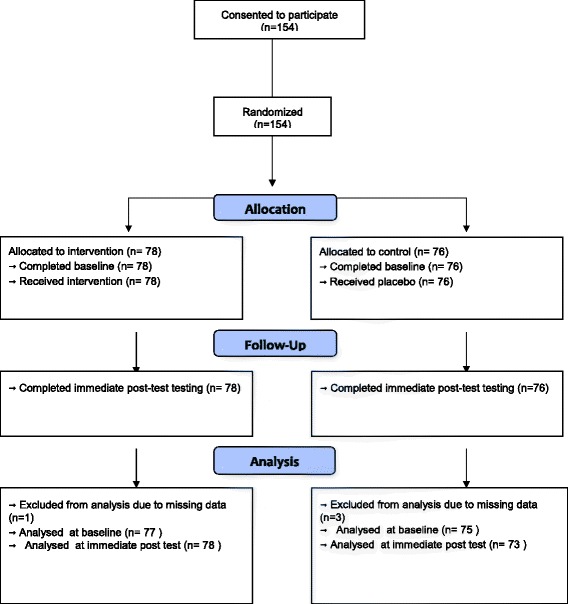
**Consort Flow Chart** **[** 42 **]** **.**

154 novices made judgements of risk of abuse (“certainty of abuse”) on the same set of 43 scenarios that the experienced clinicians had judged. Seventy-eight novices were randomised into the intervention group and 76 into the control group (see Figure 2) [42].

Of the novices, just over half - 53% (n = 81) - were medical students, 19% (n = 29) studied occupational therapy, 16% (n = 24) social work and 13% (n = 20) physiotherapy. As the novices were students on two, three or four year programmes, demographic data to their stage of training was gathered by using the generic terms of early, mid and final stage of training: 31% (n = 47) of the participants were in their early stage, 47% (n = 72) in the mid-stage of their training and 23% (n = 35) in their final stage. Age, gender and ethnicity were also noted, with 75% of novices (n = 115) < 25 years old, 25% (n = 39) > 25 years old; 71% (n = 109) being female, 29% (n = 45) male; and 66% (n = 101) reporting their ethnicity to be White, 8% (n = 12) Chinese, 6% (n = 9) Asian, 5% (n = 7) African and 16% (n = 25) of unknown ethic background.

### Did novices’ and experts’ thresholds for detection differ from each other when examining scenarios of elder financial abuse?

The experts’ mean certainty (of abuse) was higher than the novices’ mean across the first (pre intervention) 28 scenarios, indicating that they had a lower threshold for abuse identification than the novices. Here the novices in the intervention and control groups have their data amalgamated. The experts also were found to have a consistent threshold across the scenario sets i.e. their threshold remained at the same level on both scenario sets. See Table 3.

**Table 3:** **Mean certainty (of abuse) and upper 95% CI limit for pre and post intervention scenarios, and expert scores for the comparable scenarios**

**Phase**			**Students**	**Experts**
			**(n = 154)**	**(n = 33)**
Pre intervention	Mean		58.04	70.61
	95% Confidence Interval for Mean	Upper Bound	58.75	73.96
Post intervention	Mean		63.04	70.84
	95% Confidence Interval for Mean	Upper Bound	64.00	76.08

### Does training align novices’ bring detection abilities closer to that of experienced professionals?

Prior to the intervention, the control (n = 76, mean 59.15) and intervention group (n = 78, mean 56.97) did not differ significantly in the certainty assigned to their responses (Mann Whitney U = 2708, z = −.92, p > 0.05).

After the training the intervention group (n = 78) were significantly more certain of the risk of abuse (mean 64.64) than the control group (n = 76, mean 61.41), Mann Whitney U = 3598, z = 2.29, p = 0.02. See Table 4.

**Table 4:** **Certainty pre and post intervention**

	**Pre intervention certainty**			**Post intervention certainty**	
	**n**	**Mean**	**Standard deviation (SD)**	**Mean**	**SD**
Control	76	59.15	10.08	61.41	11.54
Intervention	78	56.97	10.52	64.64	10.05

### Did training bring novice detection of financial abuse risk in line with expert opinion?

Table 5 shows that the intervention group had a higher hit rate after the training (from an average 67% to 85%) but their false alarm rate also increased slightly (from 41% to 44%). Whilst the control group’s hit rate did not change between the judgements made on the first and second set of scenarios, they did make fewer false alarm judgements (See Table 6).

**Table 5:** **Hit and false alarm rates post intervention**

	**Pre intervention hit rate**		**Post intervention hit rate**		**Pre intervention false alarm rate**		**Post intervention false alarm rate**	
	**Mean**	**SD**	**Mean**	**SD**	**Mean**	**SD**	**Mean**	**SD**
Control	.69	.24	.69	.32	.44	.20	.33	.26
Intervention	.67	.27	.85	.22	.41	.20	.44	.25

**Table 6:** **Sensitivity (A**´**) and bias (C) post intervention**

	**A** **´** **pre intervention**	**A** **´** **post intervention**			**C pre intervention**		**C post intervention**	
	**Mean**	**SD**	**Mean**	**SD**	**Mean**	**SD**	**Mean**	**SD**
Control (n = 76)	.70	.13	.75	.14	-.21	.63	.09	.87
Intervention (n = 78)	.71	.11	.76	.14	-.12	.72	-.34	.63

The 78 novices receiving the intervention (mean A´, .71) were not significantly more sensitive (probability of saying “at risk” when the trial indicates “risk”) than the control group (n = 76, mean A´, .70) prior to training (Mann Whitney U = 3051, z = .32, p > 0.05). They were, however, more likely to have a tendency toward classifying scenarios as “at risk of abuse” (Mann Whitney U = 2062, z = −3.26, p = 0.001).

**Figure 3:**
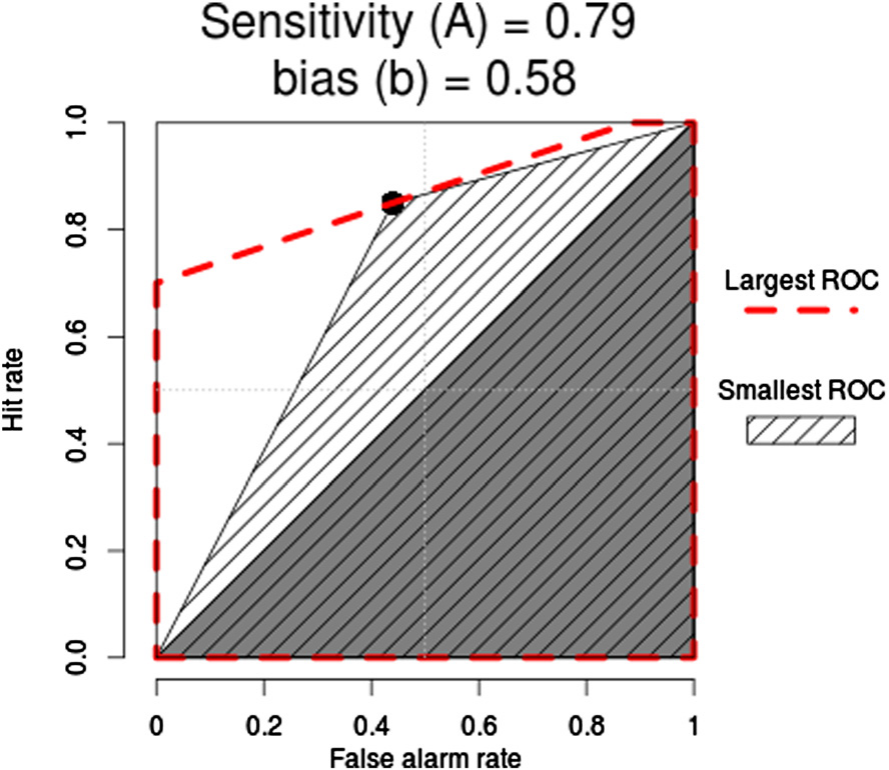
**Intervention group ROC curve.**

**Figure 4:**
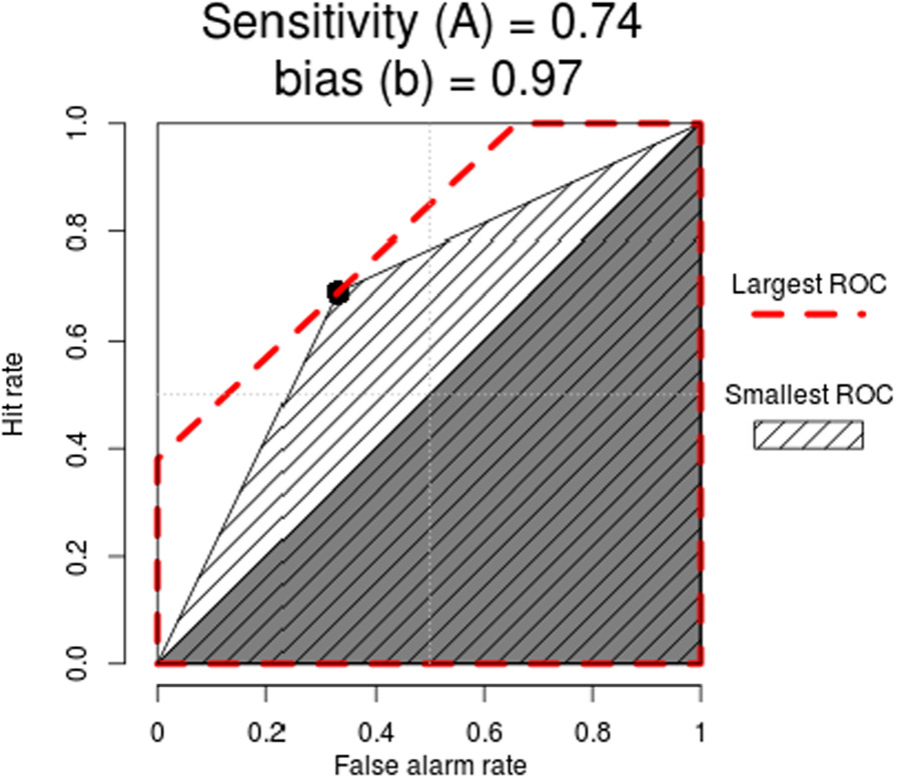
**Control group ROC curve.**

The relationship between sensitivity and bias, and the impact on hit and false alarm rates can be presented graphically using ROC curves for the intervention and control groups (See Figures 3 & 4). Note that Zhang and Mueller’s [41] ROC procedures produce a range of possible curves through a single point on the curve (in this case the point nearest to the top left of the graph). We can see that the effect of a more appropriate bias (decision tendency) in the intervention group results in better performance overall - even if their judgement sensitivity is no better than the control group.

## Discussion

Before novices had received any training, experts had a lower threshold for detection of abuse than the novices, enabling them to identify a greater number of cases of abuse. This is heartening; in an area where under reporting is rife, it is important that the more experienced professionals are more able to detect abuse than novice professionals. They can lead good practice and model effective behaviour for novices. It is also a positive that the experienced professionals remained fairly consistent in their 95% confidence interval for the mean ratings across both sets of scenarios, thereby indicating a relatively stable threshold. Of course there may have been some small variation in the level of abuse presented in the two scenario sets that could have accounted for any small degrees of variation; however the scenario sets were generated to contain relatively similar types of scenarios. Although consistency does not de facto ensure “expert” practice as experts can be consistently wrong, inconsistency is indicative of a lack of skill as it suggests that the decision maker has yet to develop a well-developed judgement policy [39]. The experts were clearly not novices in this study. In Cheyne’s [34] study, which examined midwives’ patient transfer decisions, some variation in individual decision thresholds was also found, although they did not aim to only focus on expert practice and no comparison of novice and experts’ thresholds were undertaken. The Kitchen et al. [12] findings did examine novice-expert differences in terms of identification of elder abuse and showed that novices were less able to detect abuse as compared with experts but only in clear-cut scenarios of abuse; no difference in the detection ability between novices and experts was found when judging more complex cases.

Before training, the novices were less certain whether abuse was occurring than the experts, indicating a higher threshold for classifying risk of financial abuse than the experienced professionals. After training the novices were, on average, more certain that abuse was occurring, indicating a lowering of their threshold; the training had altered the novices’ threshold with their threshold moving closer to that of the experts. This shift in their threshold enabled them to correctly identify more cases of abuse, which demonstrates enhanced skill in this context; the need being to identify under-reported cases [43].

Although the training brought the novices’ decisions more in line with those of the experts, they did not fully achieve the experts’ threshold capacity. Better signal detection ability arose from training improving novices’ ability to accurately identify cases of financial abuse by shifting their decision tendency: increasing their willingness to (appropriately) say “at risk” in the simulated cases, but also increasing false alarm rates. If translated to real practice, those who had been trained would be able to detect more cases of financial abuse than those who had not received the training, but would also risk making more unfounded accusations. This kind of trade-off is perhaps preferable to leaving more actual cases of abuse undetected.

The study is limited in that accuracy of risk assessment was measured by comparing novices’ decisions with those of nominal experts, which may indicate the potential for systematic bias. It is important to note that actual cases, with known outcomes, would provide more valid data with which to compare the accuracy of decisions. Another issue related to the degree of experience the experts had obtained. Although the sub group were identified in the original study as superior to the larger sample originally collected [37], financial abuse is not an everyday issue that professionals come across; expertise maybe more difficult to develop compared with some other types of more practised decision making with higher levels of feedback [44]. However the fact that the experts remained consistent in their decision threshold and that they exhibited the (desirable) relatively low threshold for detection of elder abuse justifies some confidence in the use of threshold as a reference standard.

The training aimed to enhance the novices’ capacity to use the case information to determine an appropriate response. However, other issues such as effective multidisciplinary working were not addressed. Such issues are seen as central to effective actions arising from abuse detection. The training also only addressed the decision needs of health and social care professionals. There may be merit in examining the signal detection ability, and responsiveness to training, of professionals involved in safeguarding and detection in areas such as the financial and legal sectors [45].

## Conclusions

Judging financial abuse risk will always be surrounded by imperfect, incomplete information and “noise”; this study has shown that training novice professionals can help. Specifically, it has shown that an educational intervention can counter a tendency to say “no risk” by shifting (appropriately) novices’ tendencies towards making “at risk” judgement calls. Whilst this study may help improve a single cohort of future professionals’ risk detection, far more work in this important area is needed in order to enhance detection expertise and to protect elders from financial abuse.

### Data availability

The data set supporting the results of this article is available in the UK Data Service ReShare repository http://reshare.ukdataservice.ac.uk/850619.

## Abbreviations

SDT: Signal Detection Theory
Consort: Consolidated standards of reporting trials
ROC: Receiver Operating Curve
